# Spectrum of phenotypic anomalies in four families with deletion of the *SHOX* enhancer region

**DOI:** 10.1186/1471-2350-15-87

**Published:** 2014-07-23

**Authors:** Valentina Gatta, Chiara Palka, Valentina Chiavaroli, Sara Franchi, Giovanni Cannataro, Massimo Savastano, Antonio Raffaele Cotroneo, Francesco Chiarelli, Angelika Mohn, Liborio Stuppia

**Affiliations:** 1 Department of Psychological, Humanities and Territory Sciences, School of Medicine and Health Sciences, “G. d’Annunzio” University of Chieti, via dei Vestini 31, 66013 Chieti, Italy; 2 Center of Excellence on Aging, “G. d’Annunzio” University Foundation, via dei Vestini 31, 66013 Chieti, Italy; 3 Department of Paediatrics, “G. d’Annunzio” University of Chieti, via dei Vestini 5, 66013 Chieti, Italy; 4 Department of Neuroscience and Imaging, Section of Diagnostic Imaging and Therapy, Radiology Division, “G. d’Annunzio” University of Chieti, Chieti, Italy

**Keywords:** Madelung deformity, MLPA, *SHOX*, Short stature

## Abstract

**Background:**

*SHOX* alterations have been reported in 67% of patients affected by Léri-Weill dyschondrosteosis (LWD), with a larger prevalence of gene deletions than point mutations. It has been recently demonstrated that these deletions can involve the *SHOX* enhancer region, rather that the coding region, with variable phenotype of the affected patients.

Here, we report a *SHOX* gene analysis carried out by MLPA in 14 LWD patients from 4 families with variable phenotype.

**Case presentation:**

All patients presented a *SHOX* enhancer deletion. In particular, a patient with a severe bilateral Madelung deformity without short stature showed a homozygous alteration identical to the recently described 47.5 kb PAR1 deletion. Moreover, we identified, for the first time, in three related patients with a severe bilateral Madelung deformity, a smaller deletion than the 47.5 kb PAR1 deletion encompassing the same enhancer region (ECR1/CNE7).

**Conclusions:**

Data reported in this study provide new information about the spectrum of phenotypic alterations showed by LWD patients with different deletions of the *SHOX* enhancer region.

## Background

*SHOX* deficiency represents a frequent cause of short stature, being associated with different pathological phenotypes such as Turner syndrome (TS), Idiopathic Short Stature (ISS; MIM ID 300582), Léri-Weill dyschondrosteosis (LWD; MIM ID 127300) and Langer mesomelic dysplasia (LS; MIM ID 249700) [1-10].

LWD is characterized by the presence of short stature associated with specific bone alterations, such as the Madelung deformity of the forearm. However, the full-blown LWD phenotype is frequently not determined in pre-schooler children because the specific features of this condition (i.e. mesomelic disproportion of the limbs and Madelung deformity), appears during the second decade of life [11-13]. As a consequence, in many cases short stature represents the only clinical sign at diagnosis. All together, mutations affecting the *SHOX* function in the different pathological conditions display an estimate frequency of less than 1:1000, thus representing the most common mendelian disease in the Caucasian population [14].

Due to this high frequency of alterations of the *SHOX* gene and to the recently demonstrated good response to the treatment with growth hormone (GH) in patients with *SHOX* deficiency, the early identification of *SHOX* alterations has become crucial for the diagnosis of the disease and the therapeutic strategy [15]. In this view, a phenotype scoring system assisting the identification of the most appropriate subjects for *SHOX* testing has been developed by Rappold et al. [16], recommending *SHOX* analysis in presence of a score greater than four out of a total possible score of 24. Moreover, Binder described an interesting algorithm approach to *SHOX* mutation screening in short children, promoting the clinical diagnosis supported by an auxological analysis of the body proportions (mesomelia), the presence of minor abnormalities, and the search for subtle radiographic signs and the molecular studies for confirming clinical data [17].

A large number of literature reports have demonstrated the presence of *SHOX* alterations in about 67% of LWD cases [18,19]. On the other hand, *SHOX* alterations are not detected in the vast majority of cases with idiopathic short stature (85–98%) [9,17,20].

The use of the MLPA assay [18-21] has disclosed that deletions can involve not only the *SHOX* coding region, but also the upstream and downstream *SHOX* enhancer sequences [22-25].

Recently, Benito-Sanz et al. [26] provided a deep characterization of a relatively small deletion of PAR1, previously reported by Chen et al. [10] and Caliebe et al. [27], uncovering a novel downstream enhancer. This deletion is correlated with a remarkably variable phenotype of patients [28], confirming the evidence that deletion size is not related with the severity of the clinical phenotype [29], which, despite the high penetrance of *SHOX* deficiency, is very variable becoming more pronounced with age and being more severe in females [17].

The identification of PAR1 deletions not involving the *SHOX* coding regions have raised novel interest to the knowledge of the mechanisms leading to short stature in cases with *SHOX* deficiency.

In order to provide a contribute to this field of studies, we report on *SHOX* gene analysis in LWD patients with selected dysmorphic signs derived from four families, all evidencing deletions of the enhancer region, which was present in homozygous form in a patient with Madelung deformity but normal stature.

## Case presentation

A written informed consent was obtained from each patient. The different techniques were performed according to standard procedures of the participating centers, with the purpose to reach a genetic diagnosis in the studied patient. It was not designed as an experimental study.

### Family 1

**Figure 1:**
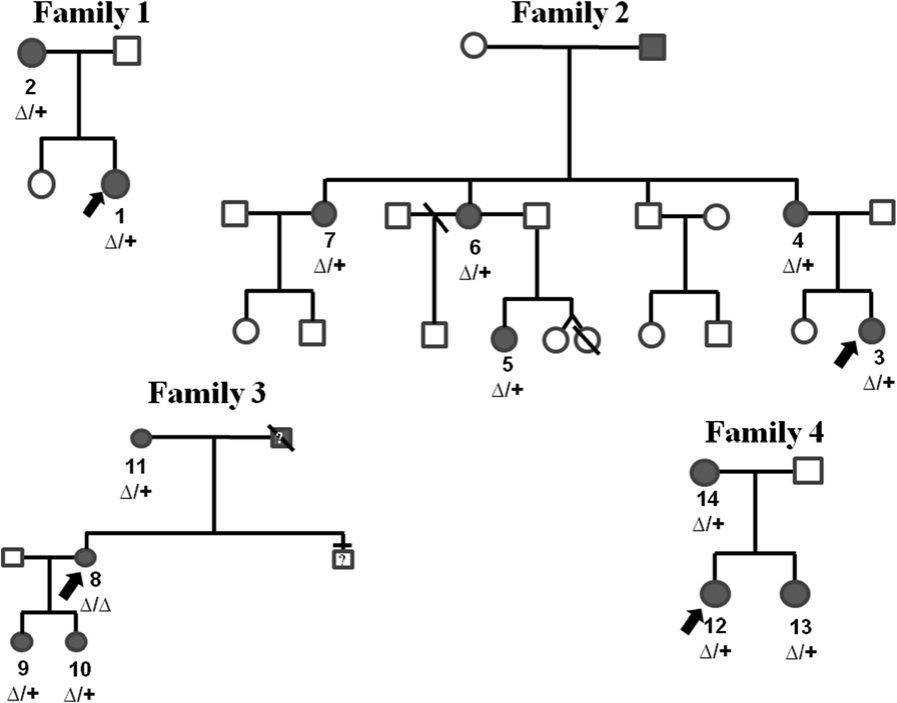
**Pedigree of the four families.** Black icon: clinically affected; white symbol: clinically unaffected; Δ: deletion; +: no mutation; ?: not tested.

The index case (Figure 1-n.1) was a 12-years and 10 months girl admitted to the outpatient Endocrine Clinic of the Department of Pediatrics, University of Chieti, Italy, for short stature. The girl was the first offspring of unrelated healthy parents. She has one 10-year-old sister, who had a normal linear and ponderal growth. The girl was born after 39 weeks of gestation after an unremarkable pregnancy. Birth anthropometric measurements were the following: weight 2.500 kg (3^th^-10^th^ percentile), length 48 cm (10^th^-25^th^ percentile). From the first months of life she had slow linear and ponderal growth, with normal psychomotor development. At our first clinical evaluation she showed short stature (140.6 cm, −2.18 SDS). Sitting height was 75 cm (−2.0 SDS), sitting height/height ratio was 0.53 (+1.0 SDS) and arm span/height ratio was 96.7%. BMI was between 25^th^-50^th^ percentile and head circumference was normal. She had mesomelia with muscular hypertrophy. No facial dysmorphism was detected except for mild webbed neck. Radiological examination of the forearms showed bilaterally slight triangular deformation of distal radial epiphysis and mild bowing of radial diaphysis; legs were normal. Bone age according to Greulich and Pyle was 13.8 yr. Based on the detected clinical and radiological signs, the total Rappold’s score was 6 (Table 1).

**Table 1:** **Clinical characteristics of 14 patients with a deletion in the downstream enhancer region of *SHOX***

**Patient**	**Age(sex)**	***Shox *** **deletion (mlpa probes)**	**Arm Span/Height ratio (<96.5%)**	**Sitting height/Height ratio (>55.5%)**	**BMI (>50°C)**	**Cubitus Valgus**	**Short forearm**	**Bowing forearm/tibia**	**Muscular hypertrophy**	**Dislocation of ulna at the elbow**	**Total score**
***FAMILY 1***											
*n.1*	12y10m (F)	heterozygous del 13296-L15336, 05645-L05099 and 05646-L15507	96.7% (0)	53% (0)	<50°c (0)	Absent (0)	Present (3)	Absent (0)	Present (3)	Absent (0)	**6**
*n.2*	49y (F)	heterozygous del 13296-L15336, 05645-L05099 and 05646-L15507	93% (2)	48% (0)	> 50°c (4)	Absent (0)	Present (3)	Absent (0)	Present (3)	Absent (0)	**12**
***FAMILY 2***											
*n.3*	3y11m (F)	heterozygous del 13296-L15336, 05645-L05099 and 05646-L15507	100% (0)	53% (0)	>50°c (4)	Present (2)	Present (3)	Absent (0)	Present (3)	Absent (0)	**12**
*n.4*	39y (F)	heterozygous del 13296-L15336, 05645-L05099 and 05646-L15507	98% (0)	41.9% (0)	>50°c (4)	Present (2)	Present (3)	Absent (0)	Present (3)	Absent (0)	**12**
*n.5*	10y (F)	heterozygous del 13296-L15336, 05645-L05099 and 05646-L15507	100% (0)	47.8% (0)	>50°c (4)	Present (2)	Present (3)	Absent (0)	Present (3)	Absent (0)	**12**
*n.6*	46y (F)	heterozygous del 13296-L15336, 05645-L05099 and 05646-L15507	98% (0)	47.2% (0)	<50°c (0)	Present (2)	Present (3)	Absent (0)	Present (3)	Absent (0)	**8**
*n.7*	48y (F)	heterozygous del 13296-L15336, 05645-L05099 and 05646-L15507	96% (2)	49% (0)	>50°c (4)	Absent (0)	Present (3)	Absent (0)	Present (3)	Absent (0)	**12**
***FAMILY 3***											
*n.8*	30y (F)	homozygous del 13296-L15336, 05645-L05099 and 05646-L15507	98% (0)	54% (0)	<50°c (0)	Present (2)	Present (3)	Present (3)	Present (3)	Present (5)	**16**
*n.9*	2y10m (F)	heterozygous del 13296-L15336, 05645-L05099 and 05646-L15507	94% (2)	56.9% (2)	<50°c (0)	Absent (0)	Present (3)	Absent (0)	Absent (0)	Absent (0)	**7**
*n.10*	11 m (F)	heterozygous del 13296-L15336, 05645-L05099 and 05646-L15507	94% (2)	58.3% (2)	<50°c (0)	Absent (0)	Present (3)	Absent (0)	Absent (0)	Absent (0)	**7**
*n.11*	54y (F)	heterozygous del 13296-L15336, 05645-L05099 and 05646-L15507	100% (0)	52% (0)	<50°c (0)	Present (2)	Present (3)	Absent (0)	Present (3)	Absent (0)	**8**
***FAMILY 4***											
n.12	14,7y (F)	heterozygous del 05645-L05099	97% (0)	50% (0)	>50°c (4)°	Present (2)	Absent (0)	Absent (0)	Present (3)	Absent (0)	**9**
n.13	11y (F)	heterozygous del 05645-L05099	97% (0)	48% (0)	>50°c (4)	Present (2)	Absent (0)	Absent (0)	Present (3)	Absent (0)	**9**
n.14	42y (F)	heterozygous del 05645-L05099	93% (2)	50% (0)	>50°c (4)	Absent (0)	Present (3)	Absent (0)	Present (3)	Absent (0)	**12**

The 49-year-old mother of the proband (Figure 1-n.2) also showed short stature (149 cm, −2.0 SDS). Sitting height was 72 cm (< −2.0 SDS), sitting height/height ratio was 0.48 cm (< −2.5 SDS) and arm span/height ratio was 93%. BMI was >50^th^ percentile. She had mesomelia with muscular hypertrophy. No facial dysmorphisms were detected except for mild webbed neck. Radiological examination of the radius and ulna showed bilateral Madelung deformity; legs were normal. The total Rappold’s score was 12 (Table 1).

### Family 2

**Figure 2:**
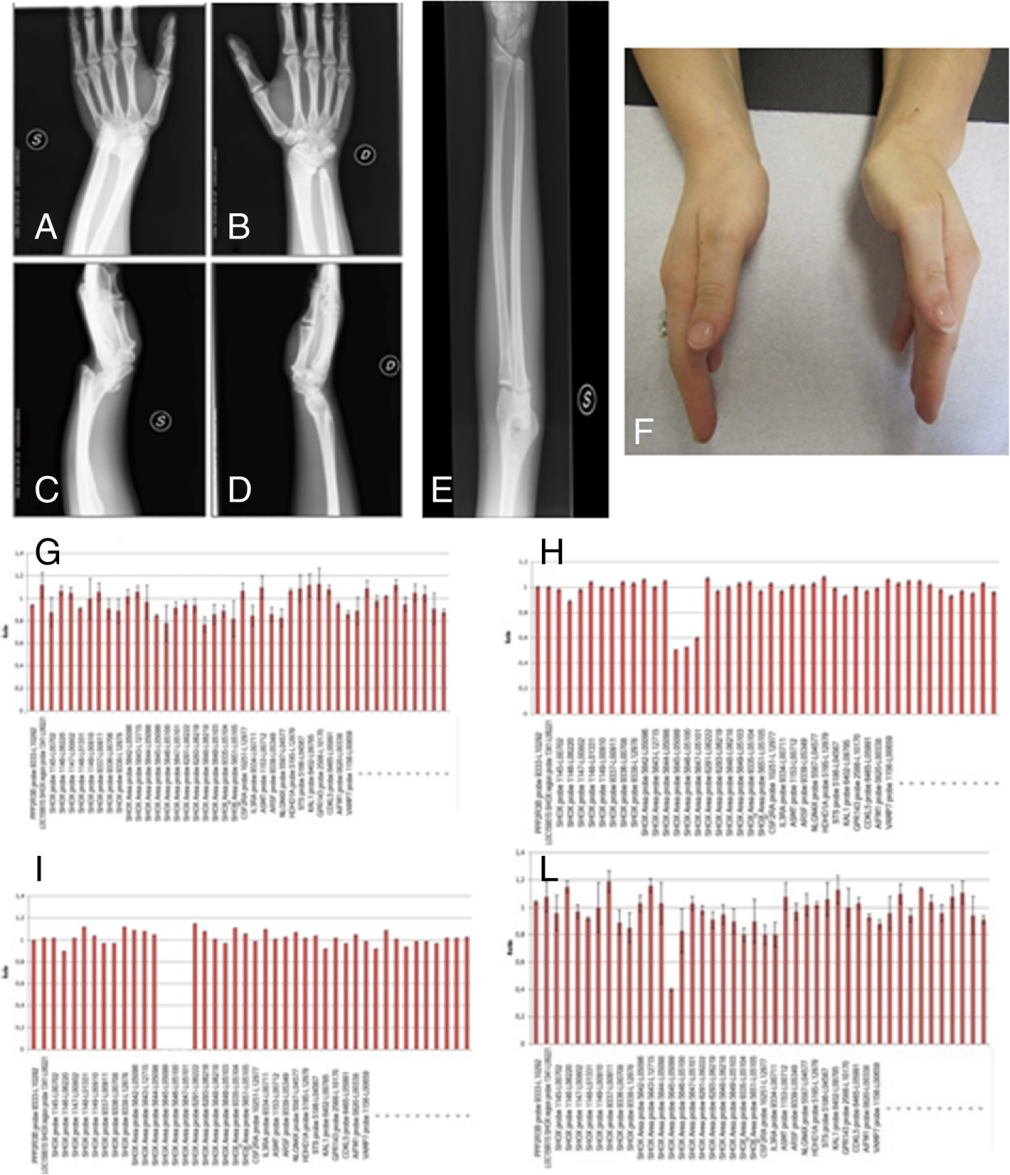
**Female 39-year-old: Bilateral forearm radiographs show triangularization of distal epiphysis and bowing of diaphysis of radius bilaterally; distal radio-ulnar articulations appeared radiographically lucent.** Palmar subluxation of the left carpus **(A-D)**. Female 6-year-old: Left forearm radiography shows triangularization of distal epiphysis and bowing of diaphysis of radius, radiolucency of distal radio-ulnar articulation **(E)**. Madelung deformity of family 3 index case (n.8) **(F)**. The lower panel showes MLPA assay results. **(G)** Control: Normal peaks were classified as showing a ratio of 0.65-1.35 **(H)** Heterozygous deletion of the 13296-L15336, 05645-L05099 and 05646-L15507. MLPA probes have a ratio < 0.65. **(I)** Homozygous deletion of the 13296-L15336, 05645-L05099 and 05646-L15507 MLPA probes showing a ratio = 0. **(L)** Heterozygous deletion of the 05645-L05099 MLPA probes.

The index case (Figure 1-n.3) was a 3-years and 11 months girl admitted to the outpatient Endocrine Clinic of the Department of Pediatrics, University of Chieti, Italy, for short stature. The girl was the second offspring of unrelated healthy parents. She has one 6-year-old healthy sister, showing normal linear and ponderal growth. The girl was born after 38 weeks of gestation after an unremarkable pregnancy. Birth anthropometric measurements were the following: weight 2.820 kg (10^th^-25^th^ percentile), length 48.5 cm (25^th^-50^th^ percentile). From the first months of life she had slow linear growth, whereas ponderal growth and psychomotor development were normal. At our first clinical evaluation she showed short stature (91.3 cm, −2.53 SDS). Sitting height was 72.6 cm (<−2.5 SDS), sitting height/height ratio was 0.53 (−1.2 SDS) and arm span/height ratio was 100%. BMI was >95^th^ percentile. The habitus was muscular, and she had mesomelia and facial dysmorphisms, such as mild hypertelorism, left epicanthus, high-arched palate, mild webbed neck, cubitus and genu valgus and lordosis. At radiological examination left radius showed a mild bowing of diaphysis, triangularization of distal epiphysis and bowing of diaphysis of radius, radiolucency of distal radio-ulnar articulation with normality of left radius, ulnas and wrists (Figure 2E); legs were normal. Bone age according to Greulich and Pyle was 3 yr. The total Rappold’s score was 12 (Table 1).

Her mother (Figure 1-n.4) (39-year-old) also showed short stature (146.4 cm, −2.43SDS). Sitting height was 61.4 cm (<−2.5 SDS), sitting height/height ratio was 41.9%and arm span/height ratio was 98%. BMI was between 50^th^-75^th^percentile. Muscular hypertrophy was detected. She had mesomelia with facial dysmorphisms including mild high-arched palate and webbed neck. Mild scoliosis with cubitus and genu valgus were detected. The patient suffered from adolescence of diffuse muscular pain, which at adult age was imputed to fibromyalgia. Radiological examination showed triangularization of distal epiphysis and bowing of diaphysis of radius bilaterally;distal radio-ulnar articulations appeared radiographically lucent and legs did not show skeletal anomalies (Figure 2A-D). The total Rappold’s score was 12 (Table 1).

A 10-year-old cousin (Figure 1-n.5) of the index case was also admitted for short stature. The girl was the second offspring of unrelated healthy parents. She has one 18-year-old healthy brother and one 7-year-old healthy sister, both of them with normal linear and ponderal growth. The girl was born after 32 weeks of gestation after an unremarkable pregnancy. Birth anthropometric measurements were the following: weight 2.850 kg (>97^th^ percentile), length 45.2 cm (90^th^-97^th^ percentile). From the first months of life she had slow linear growth with normal ponderal growth. Psychomotor development was normal. At our first clinical evaluation she showed short stature (125.3 cm, −2.07 SDS). Sitting height was 60 cm (<−2.5 SDS), sitting height/height ratio was 47.8%, arm span /height ratio was 100%. BMI was between 85^th^-90^th^ percentile. The habitus was slightly muscular, and she had mesomelia and facial dysmorphisms, including mild high-arched palate and mild webbed neck. She also had cubitus valgus and valgus knee. Bone age according to Greulich and Pyle was 8.6 yr. Radiological examination of the radius, ulnas and legs did not show significant bone alterations.The total Rappold’s score was 12 (Table 1).

Her mother (Figure 1-n.6) (46-year-old), the sister of index case’s mother, also showed mild linear impairment (149.8 cm, −1.86 SDS). Sitting height was 70.8 cm (<−2.5 SDS), sitting height/height ratio was 47.2 (<−2.5 SDS) and arm span /height ratio was 98%. BMI was between 25^th^-50^th^ percentile. The habitus was muscular, and she had mesomelia, cubitus valgus without facial dysmorphisms. The patient suffered from adolescence of diffuse muscular pain, which at adult age was imputed to fibromyalgia. Radiological examination showed bilaterally triangularization of distal epiphysis and bowing of diaphysis of radius, radiolucency of distal radio-ulnar articulation; legs were normal.The total Rappold’s score was 8 (Table 1).

Another sister of index case’s mother (Figure 1-n.7) (48-year-age old) also showed mild linear impairment (150.4 cm, −1.76 SDS). Sitting height was 75 cm (<−2.5 SDS), sitting height/height ratio was 49 (<−2.25 SDS) and arm span /height ratio was 96%. BMI was between 85^th^-95^th^ percentile. The habitus was muscular, and she had mesomelia. The patient suffered from adolescence of diffuse muscular pain, which at adult age was imputed to fibromyalgia. Radiological examination of the radius and ulna showed bilateral Madelung deformity. The total Rappold’s score was 12 (Table 1).

### Family 3

The index case (Figure 1-n.8) was a 30-year-old women admitted to the outpatient Genetic Clinic, University of Chieti, Italy, for Madelung deformity surgically corrected at the age of 18 (Figure 2F). The women was the second offspring of unrelated healthy parents. She has one 26-year-old healthy brother, who had a normal linear and ponderal growth. The women was born at term after an unremarkable pregnancy. Birth anthropometric measurements were in the normal rage. From the first months of life she had slow linear growth with normal ponderal growth and psychomotor development. At our first clinical evaluation she did not show short stature (155.6 cm, −0.89 SDS). Sitting height was 84 cm (< −2.5 SDS), sitting height/height ratio was 54% (< −2.5 SDS) and arm span/height ratio was 98%. BMI was between 25^th^-50^th^ percentile. The habitus was muscular, and she had mesomelic, facial dysmorphisms (hypertelorism) and cubitus and genu valgus with scoliosis. The patient suffered from adolescence of diffuse muscular pain. Radiological examination of the radius and ulna showed bilateral Madelung deformity (Figure 2F). The total Rappold’s score was 16 (Table 1).

This patient had two daughters. The first girl (Figure 1-n.9) was born after 37 weeks of gestation after an unremarkable pregnancy. Birth anthropometric measurements were the following: weight 2.560 kg (10^th^-25^th^percentile), length 49.5 cm (75^th^-90^th^ percentile). She showed decrease of linear growth from the age of 21 months, while poor ponderal growth was detected from the age of 1 year. Psychomotor development was normal. At our first clinical evaluation she was 2-years and 10 months old. She showed short stature (87 cm, −2.09 SDS). Sitting height was 49 cm (<−2.5 SDS), sitting height/height ratio was 56.9% (<−2.0 SDS) and arm span/height ratio was 94%. BMI was at 10^th^ percentile. Head circumference was normal. The habitus was not muscular, and she had mesomelia and facial dysmorphisms, including hypertelorism, epicanthus and mild webbed neck. Bone age according to Greulich and Pyle was 2.5 yr. Radiological examination of the forearms was normal.The total Rappold’s score was 7 (Table 1).

The second girl (Figure 1-n.10) was born after 37 weeks of gestation after an unremarkable pregnancy. Birth anthropometric measurements were the following: weight 2.430 kg (3^th^-10^th^ percentile), length 46 cm (10^th^-25^th^ percentile). She showed poor linear and ponderal growth from the age of six months. Psychomotor development was normal.At our first clinical evaluation she was 11-month-old. She showed short stature (72 cm, −4.11 SDS). Sitting height was 42 cm (< −2.5 SDS), sitting height/height ratio was 58.3% (<−2.5 SDS) and arm span/height ratio was 94%. BMI was at 10^th^ percentile. Head circumference was normal.The habitus was not muscular. She did not show mesomelia but facial dysmorphisms were detected, including epicanthus, hypertelorism and mild webbed neck. Bone age according to Greulich and Pyle was 1 yr. Radiological examination of the radius and ulna was normal.The total Rappold’s score was 7 (Table 1).

The index case’s mother (Figure 1-n.11) (54 year-old) did not show short stature (159 cm, −0.32 SDS). Sitting height was 75 cm (<−2.5 cm), sitting height/height ratio was 52% (<−2.5 SDS) and arm span/height ratio was 100%. BMI was between 25^th^-50^th^ percentile. The habitus was muscular, and she had mesomelia with facial dysmorphisms (hypertelorism) and mild scoliosis. Radiological examination of the radius and ulna showed bilateral Madelung deformity, with left ulnar dorsal dislocation. The total Rappold’s score was 8 (Table 1).

### Family 4

The index case (Figure 1-n.12) was a 14-years and 7 months girl admitted to the outpatient Endocrine Clinic of the Department of Pediatrics, University of Chieti, Italy, for short stature. The girl was the first offspring of unrelated healthy parents. She was born after 40 weeks of gestation after an unremarkable pregnancy. Birth anthropometric measurements were the following: weight 3.180 kg (25^th^-50^th^ percentile), length 48 cm (10^th^-25^th^ percentile). From the first months of life she had slow linear and ponderal growth, with normal psychomotor development. At our first clinical evaluation she showed short stature (147.6 cm, −1.79 SDS). Sitting height was 75 cm (−2.5 SDS), sitting height/height ratio was 0.50 (−2.0 SDS) and arm span/height ratio was 97%. BMI was between 50^th^-75^th^ percentile and head circumference was normal. She had mild webbed neck, muscular hypertrophy, pectus carenatum, and cubitus and genu valgus. Radiological examination of the radius and ulna showed bilateral Madelung deformity. Bone age according to Greulich and Pyle was 16 yr. Based on the detected clinical and radiological signs, the total Rappold’s score was 9 (Table 1).

The sister of the proband (Figure 1-n.13) (11-year-old) showed a normal linear growth (138 cm, −1.0 SDS), sitting height was 67 cm (< −2.5 SDS), sitting height/height ratio was 0.48 cm (< −2.5 SDS) and arm span/height ratio was 97%. BMI was >85^th^ percentile. She had muscular hypertrophy, pectus carenatum, and cubitus and genu valgus. Radiological examination showed bilaterally triangularization of distal epiphysis and bowing of diaphysis of radius, correlated to Madelung deformity. Bone age according to Greulich and Pyle was 12 yr. The total Rappold’s score was 9 (Table 1).

The mother of the proband (Figure 1-n.14) (42-year-old) also showed normal stature (160.2 cm, −0.12 SDS). Sitting height was 80 cm (< −2.5 SDS), sitting height/height ratio was 0.50 cm (< −2.0 SDS) and arm span/height ratio was 93%. BMI was >95^th^ percentile. She had mild mesomelia with muscular hypertrophy. No facial dysmorphisms were detected except for webbed neck. The patient suffered from adolescence of diffuse muscular pain.Radiological examination of the radius and ulna showed bilateral Madelung deformity. The total Rappold’s score was 12 (Table 1).

Laboratory investigations allowed us to exclude thyroid dysfunction, abnormal IGF-1 levels and celiac disease in all the investigated patients, which all had normal diploid karyotype.

### Molecular analysis

Genomic DNA was extracted from peripheral blood or buccal swab by QIAamp DNA Blood Midi Kit (Qiagen, Hilden, Germany). *SHOX* deletions were detected using the MRC-Holland MLPA kit (Salsa P018-E and F1; MRC-Holland, Netherlands – Resnova, Italy) according to the manufacturer’s instructions. The P018-D1 *SHOX* probemix contains 44 MLPA probes with amplification products between 130 and 463 nt. Seven of these probes are specific for each exon of the human *SHOX* gene and one is mapped just before the *SHOX* promoter region (4 kb before *SHOX*-PAR1). In addition, 14 probes are present detecting sequences in a region downstream of *SHOX* which has been implicated in regulation of *SHOX* transcription. Furthermore, nine probes on the X chromosome, out of PAR regions, are included in this probemix. Finally, ten autosomal reference probes are included. All the patients were also investigated with the last MLPA *SHOX* probemix (P018-F1) that as compared to oldest D1version three new probes near the PAR1 boundary have been included. Two probes (GPR143 and 13296-L15336) has been removed. The 88 and 96 nt control fragments have been replaced (QDX2) (http://www.mrc-holland.com). Data Analysis was performed using the Coffalyser software v. 9.4 (http://coffalyser.wordpress.com/).

In order to rule out false positive cases due to the presence of polymorphisms hampering the MLPA probe, we further investigated cases with deletion using PCR amplification with primers mapped within the probes region (*SHOX*_318 F: ACACCCAGTCATGAATGCAA; *SHOX*_318 R: CTTGGCTGGACAGACTCAGG; *SHOX*_432 F: ACATCGGCCTTTCCAAATAA; *SHOX*_432R: CTCGGGAGGCAGAGAGATTT), followed by direct sequencing on ABI 3130XL (ABI, Warrington, UK).

### Results

MLPA analysis, carried out in the four index patients and their familiars (N = 10), evidenced a heterozygous deletion of probes 13296-L15336, 05645-L05099 and 05646-L15507 (47543 bp deletion) in nine patients (patients 1–7, 9, 10) and a homozygous deletion of the same probes in the patient n. 8 (Figure 2H, I). A heterozygous deletion encompassing only the probe 05645-L05099 was revealed in three related patients (patients 12–14) (Figure 2L). All detected deletions involved a distinct *SHOX* enhancer region, not affecting the *SHOX* coding region.

All the data were confirmed using the latest MLPA *SHOX* probemix (P018-F1). Since in this last probe mix version the probe 13296-L15336 was removed because not reliable, the 47543pb deletion was characterized by the absence of the remaining two MLPA probes 05645-L05099 and 05646-L15507.

MLPA results were confirmed by PCR and DNA sequencing, showing absence of amplification in the patient with a homozygote deletion and absence of polymorphisms in all the observed heterozygous deletions.

### Discussion

The *SHOX* gene belongs to a family of transcriptional regulators that are mainly expressed in the middle portion of the limbs where assure the correct balance between proliferation and apoptosis during bone development. The absence of wild type *SHOX* would promote atypical proliferation of the chondrocytes combined with defective differentiation, leading to retarded longitudinal bone growth [30,31]. This specific pattern of expression can explain the wide phenotypic variability of *SHOX* deficiency patients with cases of normal stature but mesomelia and Madelung deformity.

In the present study we investigated *SHOX* region molecular defect in four LWD families, three of which showed the recurrent ~47.5 kb PAR1 deletion, previously described by Benito-Sanz et. al [26]. This deletion is mapped downstream of the *SHOX* gene and contains an enhancer sequence (ECR1/CNE7). The deletion was present in heterozygous state in all the analyzed members except one case of homozygous deletion, which surprisingly presented severe bilateral Madelung deformity but a normal stature within her target height range.

The fourth analyzed family is of particular interest, since we found, for the first time, a heterozygous deletion encompassing only one of the three classical MLPA probes characterizing the recurrent – 47.5 kb PAR1 deletions. The rearrangement cosegregated with the LWD phenotype in all the members of the family, with a clinical phenotype similar to the one showed by cases with larger deletion, confirming a pathogenic effect also of this shorter enhancer deletion. Looking in detail at this group of 14 patients it appears evident that the phenotype of patients with deletions in the 3’-PAR1 region is remarkably variable and not related to the extension or the homozygous and heterozygous form. Up to date only a few studies have been performed concerning the phenotype showed by patients carrier of enhancer deletions, all suggesting a great variability [7,28]. Kant et al. described a case in which the enhancer deletion was associated with normal stature, although below the target height range [28].

In order to better assess the clinical expression in our cohort, we calculated the Rappold score of the investigated patients [16]. Interestingly, all patients had a score greater than 4 with a median value of 11.5 (range 6–13), but a great variability was found among the different clinical signs. In fact, short forearm and muscular hypertrophy were the two most observed dysmorphic abnormalities (100% and 80%, respectively), while cubitus valgus and BMI greater than 50^th^ percentile were less frequent (60% and 40%, respectively). Unexpectedly, arm span/height ratio and sitting height/height ratio were uncommon (30% and 20%, respectively), whereas bowing of forearm and tibia as well as dislocation of ulna at the elbow were present only in one subject (Table 1).

The results obtained from the study of these four families suggest that, even if all the patients were eligible for the *SHOX* molecular analysis, some of the Rappold criteria are not very distinctive for *SHOX* deficiency. In fact, we had to take into account other characteristics, such as Madelung deformity, found in 60% of our sample. Therefore, it could be speculated that mutations in the *SHOX* enhancer region seem to be responsible mainly for bone deformities. In fact, both the homozygous case and her mother, who presented a heterozygous mutations in the *SHOX* enhancer region, showed bilateral Madelung deformity but not short stature.

The explanation of these findings probably is correlated with the etiology of the Madelung deformity, which originates with disorganized growth of part of the radial epiphysis, leading to radial bowing, premature fusion of that epiphysis, dorsal dislocation of the ulna, and wedged carpal bones. The premature fusion of the physis leads to cessation of longitudinal growth, and is always located in the ulnar zone of the distal radius but varies in the antero-posterior plane [32].

According to literature data, in our cohort, we did not find a correlation between bone defect, gender and the deletion size. These could be related to estrogens influence on the growth plate, which worse dyschondrosteosis leading to severe bone pain [33]. In this respect, it is interesting to underline that in this report many patients suffered from chronic widespread pain and received diagnosis of fibromyalgia. Since the pathogenesis of this musculoskeletal disorder is still unknown, a potential role of *SHOX* gene could be hypothesized, and further studies are required to clarify the relationship between *SHOX* deficit and fibromyalgia. In addition there isn’t an age-dependent phenotype in these subjects.

## Conclusions

In conclusion, the present report confirms the usefulness to perform *SHOX* analysis including the enhancer sequence in patients with elevated Rappold’s score. In addition, it could be useful to follow-up these patients in order to verify the onset of fibromyalgia or other chronic idiopathic musculoskeletal disorders. Finally, the detection of a case of homozygous deletion of this region in a patient with severe bilateral Madelung deformity but a normal stature suggests the role of *SHOX* gene enhancer in contributing to different anomalies, which constitutes a wide spectrum of the disease. Therefore, the *SHOX* deficiency represents a complex and heterogeneous group of conditions ranging from the more severe phenotype (Langer syndrome) to milder forms (isolated short stature/isolated Madelung deformity).

### Consent

Written informed consent was obtained from the patient for publication of this Case report and any accompanying images. A copy of the written consent is available for review by the Editor of this journal.

## Abbreviations

*SHOX*: Short stature homeobox; PAR1: Pseudoautosomal Region 1; TS: Turner syndrome; ISS: Idiopathic short stature; LWD: Léri-Weill dyschondrosteosis; LS: Langer mesomelic dysplasia; SDS: Standard deviation score; GH: Growth hormone; FISH: Fluorescence in situ hybridization; MLPA: Multiplex ligation-dependent probe amplification; BMI: Body mass index; DNA: Deoxyribonucleic acid.

## Competing interests

The authors declare that they have no competing and non-financial interests and also reveal any non-financial competing interests.

## Authors’ contributions

VG: contributed to conception and design, MLPA data analysis and drafted the manuscript. CP: responsible of clinical, participated in its design and coordination and drafted the manuscript. VC: responsible of clinical data and participated in its design and coordination. SF: performed the *SHOX* gene study through MLPA. GC, MS and ARC: responsible of radiographic studies. AM: responsible of clinical data and participated in its design and coordination. FC: helped to draft the manuscript. LS: have given final approval of the version to be published. All authors read and approved the final manuscript.

## Pre-publication history

The pre-publication history for this paper can be accessed here:

http://www.biomedcentral.com/1471-2350/15/87/prepub
